# Post-Intensive Care Syndrome as a Burden for Patients and Their Caregivers: A Narrative Review

**DOI:** 10.3390/jcm13195881

**Published:** 2024-10-02

**Authors:** Giovanni Schembari, Cristina Santonocito, Simone Messina, Alessandro Caruso, Luigi Cardia, Francesca Rubulotta, Alberto Noto, Elena G. Bignami, Filippo Sanfilippo

**Affiliations:** 1 School of Anaesthesia and Intensive Care, University “Magna Graecia”, 88100 Catanzaro, Italy; giovannischembari49@gmail.com; 2 Department of Anaesthesia and Intensive Care, A.O.U. “Policlinico-San Marco”, 95123 Catania, Italy; cristina.santonocito@gmail.com (C.S.); messina.simone05@gmail.com (S.M.); alessandrocaruso2892@gmail.com (A.C.); 3 Department of Human Pathology of Adult and Childhood “Gaetano Barresi”, University of Messina, 98124 Messina, Italy; luigi.cardia@unime.it (L.C.); alberto.noto@unime.it (A.N.); 4 Department of Surgery and Medical-Surgical Specialties, Section of Anesthesia and Intensive Care, University of Catania, 95123 Catania, Italy; frubulotta@hotmail.com; 5 Division of Anesthesia and Intensive Care, Policlinico “G. Martino”, 98124 Messina, Italy; 6 Anesthesiology, Critical Care and Pain Medicine Division, Department of Medicine and Surgery, University of Parma, 43100 Parma, Italy; elenagiovanna.bignami@unipr.it

**Keywords:** critical care, critical illness, mechanical ventilation, rehabilitation, recovery

## Abstract

Millions of critically ill patients are discharged from intensive care units (ICUs) every year. These ICU survivors may suffer from a condition known as post-intensive care syndrome (PICS) which includes a wide range of cognitive, psychological, and physical impairments. This article will provide an extensive review of PICS. ICU survivors may experience cognitive deficits in memory and attention, with a slow-down of mental processing and problem-solving. From psychological perspectives, depression, anxiety, and post-traumatic stress disorder are the most common issues suffered after ICU discharge. These psycho-cognitive impairments might be coupled with ICU-acquired weakness (polyneuropathy and/or myopathy), further reducing the quality of life, the ability to return to work, and other daily activities. The burden of ICU survivors extends to families too, leading to the so-called PICS-family (or PICS-F), which entails the psychological impairments suffered by the family and, in particular, by the caregiver of the ICU survivor. The development of PICS (and PICS-F) is likely multifactorial, and both patient- and ICU-related factors may influence it. Whilst the prevention of PICS is complex, it is important to identify the patients at higher risk of PICS, and clinicians should be aware of the tools available for diagnosis. Stakeholders should implement strategies to achieve PICS prevention and to support its effective treatment during the recovery phase with dedicated pathways and supporting care.

## 1. Introduction

Intensive care units (ICUs) are specialized hospital wards where critically ill patients receive close monitoring and specialized medical care. ICU environments strive to enhance patient outcomes, reduce mortality rates, and promote a high quality of care. However, the substantial improvements in ICU survival rates seen recently are counterbalanced by an increase in the burden of financial costs and the physiological and psychological issues of recovering ICU survivors [1]. Indeed, there is a growing amount of studies in the literature on how ICU survivors may suffer from heavy impairments in their physical, cognitive, and mental health [1,2,3]. The ensemble of all these conditions is known as “post-intensive care syndrome” (PICS) [4]. Further, a profound impact on the ICU caregivers has also been demonstrated [5], configuring the so-called PICS-F, where the latter letter stands for “family” to indicate the repercussions on the closest family members of the recovering ICU patient. Despite PICS being a complex area that also involves patients’ families, in this review, we aim to describe PICS which has a profound impact on critically ill adults discharged alive from the ICU and on their families. Despite our attempt to be comprehensive, this review is narrative and subject to errors due to a non-systematic approach in the literature search.

## 2. History of PICS and the Identification of Patients at Risk

PICS was described for the first time during the Critical Care Congress conference convened by the Society of Critical Care Medicine (SCCM) in September 2010 [6]. The main purpose of this new definition/syndrome was to increase stakeholders’ awareness and understanding of long-term outcomes after critical illness of both patients and their families, and how the continuum of care after discharge from the hospital could be shaped and improved. Accordingly, the term PICS refers to “new or worsening impairments in physical, cognitive, or mental health status arising after critical illness and persisting beyond acute care hospitalization”. In this “syndrome”, there is a possible involvement of three fields of health and well-being, namely the psychological, the cognitive, and the physical aspects [6]. Notably, over the years, the concept of PICS has become larger, including “social rehabilitation” after ICU discharge as well [7].

The range of risk factors that make ICU survivors vulnerable to developing PICS seems large. During the International Consensus Conference of the SCCM on the Prediction and Identification of Long-Term Impairments After Critical Illness [8], the attendees tried to define the main risk factors for PICS. The main aim was to preemptively identify those who are most likely to develop impairments in cognition, mental health, or physical health after ICU stays. These factors could be summarized as follows:

- Temporally antecedent to the ICU stay itself and linked to the patients themselves (age, gender, pre-existing comorbidities, psychological impairment, etc.);
- Related to the cause of admission for critical illness (sepsis or shock);
- Associated with the development of complications during the ICU stay (i.e., prolonged and deeper sedation, protracted mechanical ventilation, or delirium);
- Subsequent to the ICU discharge (early symptoms of anxiety or depression) [8].

In a recent meta-analysis, Lee and al. identified at least 60 individuals’ risk factors for PICS, dividing them into patient-related (social demographics, personality, and previous health) and ICU-related (ICU admission and experience during the ICU stay) factors [9]. Such factors are summarized in Table 1 [9].

Among the patient-related factors, some appear to be more relevant than others. Women seem more inclined to develop psychological symptoms after ICU discharge [10]. Age seems to play a key role too, with older patients having a higher incidence of PICS as compared to younger adults. Moreover, a higher education status may act as a protective factor [11]. Certainly, ICU-related factors are fundamental contributors to the occurrence of PICS. According to the Bringing to Light the Risk Factors and Incidence of Neuropsychological Dysfunction in ICU Survivors (BRAIN-ICU) study, the length of hospitalization is directly related to poorer cognitive function tests at three and twelve months after ICU discharge [12]. Two studies confirmed the importance of ICU length of stay on the incidence of PICS. In particular, Herridge et al. reported the 1-year outcome in patients exposed to at least 7 days of MV, and stratified patients into four disability groups. The authors suggested that two factors, age older than 66 years and ICU stay greater than 2 weeks, were associated with the worst disability and with a 40% mortality at 1-year follow-up [13]. Similarly, Needham et al. evaluated survivors previously admitted to the ICU with acute lung injury; in their analysis accounting for baseline conditions, the authors reported a significant association and interaction between the daily dose of corticosteroids and the ICU stay on one side, with physical impairments at 6 and 12 months [14]. 

The need to use lifesaving treatments like mechanical ventilation (MV), the application of physical restraints, and the use of prolonged and deeper sedation have also been reported as strong predictors for PICS [15]. Interestingly, in a single center study, Jones et al. found higher levels of anxiety (2 weeks after ICU discharge) and PTSD-related symptoms and panic attacks (at 8 weeks) in patients reporting delusional memories who had no factual recall of their ICU stay. This result may suggest that even relatively unpleasant recalls of real events during ICU stay may offer some protection from anxiety and/or subsequent PTSD-related symptoms [16]. Patients experiencing agitation and delirium during their ICU stay seem more likely to develop long-term cognitive impairment [17]. Moreover, the severity of the illness leading to ICU admission seems relevant to the development of PICS symptoms. It appears that over half of patients with severe illnesses such as sepsis, acute respiratory distress syndrome (ARDS), and multi-organ failure experience one or more PICS symptoms at 3 and 12 months [11,18]. The assessment of the patient’s conditions prior to the ICU admission may help to identify those at increased risk, although these factors should be contextualized with those arising after the ICU admission and the necessary treatments. Such evaluation may help clinicians to develop a better understanding and prediction of the PICS, thus carefully focusing on prevention and early support. Whilst more studies are desirable to identify risk factors and also to weight their specific impact on PICS development, the implementation of proper support for patients experiencing PICS remains a challenge considering the actual projection of financial constraints and the healthcare staff shortage.

## 3. Clinical Manifestations of Pics: Multi-Dimensional Impairments

The clinical manifestations associated with PICS are related to the impairment of three big areas of individual health: physical, cognitive, and mental. Moreover, very often, more than one of these is affected [11,19]. Hereby, for didactic purposes, we separately discuss the impairments at the physical, cognitive, and psychological level.

### 3.1. Physical Dysfunction

Under the umbrella of physical dysfunction, ICU-acquired weakness (ICUAW) plays a central role in patients recovering after critical illness. In particular, critical illness polyneuropathy (CIP) and/or myopathy (CIM), clinically defined by total score of the Medical Research Council (MRC) scale below 48 points, are the two pathogenic hallmarks of ICUAW [20]. ICU-AW is typically generalized, symmetrical, and affects the proximal limbs more commonly than the distal limbs; moreover, it impairs the function of respiratory muscles, consequently affecting the respiratory weaning, whilst the facial and the ocular muscles are usually spared. Such weakness may be of neurogenic, myogenic or combined origin; in the latter case, it is also labeled as critical illness neuromyopathy [21]. ICUAW has an impact both on short- and long-term outcomes. In fact, the occurrence of ICUAW prolongs further the ICU stay, complicates the weaning process and increases the duration of MV, finally producing a negative impact on in-hospital mortality and on survival after hospital discharge [22,23]. One of the main issues related to post-ICU physical dysfunction is its detrimental effect on patient independence after discharge. Yende et al. showed that, among patients living independently before their ICU admission, almost half of them lost such ability 6 months after ICU discharge [24]. Several studies have shown that ICU survivors may exhibit muscle weakness (forced and aerobic capacity reduction), which persists for up to 5 years with a relevant impact on their quality of life (QOL); such a finding is more common in patients admitted for ARDS and/or septic shock [25,26,27]. Several drugs have also been considered as facilitators of the development of ICUAW, namely steroids and muscle relaxants. However, their relationship with ICUAW is not completely clear [28]. Although ICUAW is a significant issue for the patients recovering from ICU and consequently a burden for their families, other pathologies are also part of the umbrella of physical dysfunction associated with PICS. Among these are heterotopic ossification [29], dysphagia [30] and sexual dysfunction. 

The recovery rate after ICU discharge seems variable [31] and the current diagnostic tools do not seem sensitive enough to predict a patient’s healing. The questionnaires exploring the QOL of patients discharged from the ICU aim to evaluate their long-term outcomes. Despite being valuable tools, such questionnaires may not fully account for all the important aspects from the patient’s perspective. For example, the return to sexual activity is not explored, despite such an issue representing a real problem for the patients, and consequently for their partners. For instance, Ulvik et al. found that around one-third of ICU trauma patients were still affected by impaired sexual function three years after their injury. Similarly, the authors found that erectile dysfunction represented a real problem in males below 40 years discharged from the ICU when compared to a control group of peers not admitted to the ICU [32]. On top of the risk factors such as ICU admission and prolonged ICU stay, it must be considered that some etiological agents such as SARS-CoV-2 pose additional risks for the development of sexual impairment [33,34]. It seems reasonable that, in order to gather a full understanding of the QOL, sexual functioning should also be included when evaluating long-term outcomes after an ICU stay.

### 3.2. Cognitive Dysfunction

Cognitive dysfunction refers to a series of deficits regarding different aspects such as memory, attention, speed of mental processing, speaking and problem-solving. These deficits finally result in the inability to function normally in everyday life and subsequently produce a low health-related quality of life (HRQOL). The BRAIN-ICU study evaluated the cognitive capabilities of ICU survivors 3 and 12 months after their ICU discharge [3]. These patients showed impaired global cognition scores, with 40% of them presenting scores 1.5 standard deviations below the average of the population 3 months after ICU discharge, and also around one-quarter of ICU survivors had scores comparable to patients with mild Alzheimer’s disease [35]. For instance, it has been shown that ICU patients hospitalized for sepsis are more likely to develop moderate/severe cognitive impairment at one year as compared to non-septic patients [36]. The main hypothesis about the physio-pathological basis of the cognitive dysfunction is that sepsis, and the subsequent critical-illness-associated systemic inflammation, can induce some acute central nervous system injury damaging the blood–brain barrier endothelial cells or the neuroglial cells, therefore causing a low-grade long-lasting inflammation [37]. Among the potentially modifiable risk factors for cognitive dysfunction, Sakusik et al. reported the following: delirium, prolonged MV, presence of hypoxia and glycemic alterations, use of psychotropic medications, blood pressure instability, and transfusion with blood and blood products [38]. Delirium is certainly one of the most studied risk factors. A longer duration of delirium seems associated with worse global cognition and executive function scores at 3 and 12 months, with a robust association between the delirium severity/duration and long-term cognitive decline [39,40]. In support of this hypothesis, in a cohort of ICU survivors, Morandi et al. found a positive relationship between the duration of delirium during ICU stay and the white matter disruption seen in magnetic resonance imaging, suggesting that modern neuroimaging techniques may help the screening of patients at risk of post-ICU cognitive decline [41]. These studies suggest the value of introducing tools to detect brain frailty in daily clinical practice and to reinforce the need to prevent delirium during ICU stay. However, a pharmacological intervention clearly reducing the incidence of delirium in the ICU has not yet been found. Mortensen et al. evaluated the effect of haloperidol in patients admitted to ICU and developing delirium. Although they found a reduced mortality at 1-year follow-up, the effects on HRQOL were statistically negligible [42]. Other drugs like melatonin and its analog ramelteon have been evaluated for their impact on the prevention of delirium in ICU patients, but they do not seem to offer advantages [43].

### 3.3. Psychological Dysfunction

Mental health impairment is very common and negatively affects the HRQOL of ICU survivors. The most common psychological problems encountered are depression, anxiety, and post-traumatic stress disorder (PTSD). The incidence of these mental health disturbances is very variable, of course depending also on the cohort of ICU patients studied. Indeed, the type of ICU, the patient’s comorbidities, the reason for admission and the complications during the ICU stay will largely influence the subsequent risk of developing a psychological dysfunction after the ICU discharge. Moreover, both the incidence and the magnitude of mental health issues are related to the screening tool used for the diagnosis and the cut-offs adopted, with significant variability among studies [1,44].

The percentage of survivors manifesting depressive symptoms after ICU discharge ranges between 25% and 60% [45,46,47]. In this regard, a significant association has been highlighted between the development of depressive symptoms and the patient’s gender, with an increased risk in females. A recent meta-analysis by Lee et al. suggested that females are more prone to develop both mental health issues (odds ratio 3.4) and physical impairment (odds ratio 2.0) as compared with males [9]. Such symptoms should not be underestimated since they are associated with higher social costs, with a decrease in the HRQOL and an increased risk of suicide. Unsurprisingly, the identification of a depressive mood prior to ICU admission is a hint for the development of depressive symptoms after discharge, for longer hospitalization and for greater physical disability [48,49]. 

The number of patients experiencing anxiety after ICU discharge lies again in a very variable range, from 16% to 62% [44]. Memories of delusional events and psychiatric symptoms during hospitalization are risk factors for the future development of anxiety symptoms [50]. Interestingly, as demonstrated by a multicenter study, PTSD and anxiety are often associated, with the former usually manifesting after the latter rather than presenting as an isolated entity [51]. 

The third type of psychological dysfunction after ICU discharge is represented by PTSD. It takes about a year for ICU survivors to develop PTSD [52], and a recent meta-analysis suggests an overall prevalence of PTSD of around 25% in patients discharged from ICU, a figure comparable to survivors of military conflicts [53]. However, the estimates vary among different studies (ranging from 4% to 62%), suggesting again heterogeneity in its assessment [50,54]. Notably, a systematic review suggests a significant impact of PTSD on the patient’s QOL, since PTSD can persist for up to eight years in 24% of the cases [10]. In order to gain a better understanding of the evolution of PTSD over time, in a cohort of sepsis survivors, Konrad et al. identified three main trajectories resulting in the following clusters: (1) stable with low degree of symptoms; (2) initially severe symptoms followed by remission; (3) worsening with progressive increase in symptoms [55]. In a two-year follow-up study on ICU survivors, Bienvenu et al. identified some patterns in PTSD symptom evolution: no symptoms, “maintainers”, “remitters” and “relapsers” [54]. As compared to traditional PTSD, it seems that PTSD resulting from ICU admission and treatments may be characterized by a more delayed onset, with a progressive worsening during the second year of follow-up [55,56]. Several risk factors have been identified as facilitators of PTSD: traumatic memories during ICU stay, duration of sedation, opioid dosage, nightmares, feeling breathless, delirium, benzodiazepine dose, pre-existing depression and anxiety disorder, lower education level, alcohol abuse, and female sex [10,50,57]. In different studies, female gender has been associated with an increased risk of PTSD. The study of van Zuiden et al. that focused on PTSD trajectories based on gender differences seems of particular interest, demonstrating that females are more likely to recover while men are prone to manifest symptoms with delayed presentation [58]. This is in contrast to the findings of Konrad et al. and Lowe et al., showing that females are at higher risk of both early and late post-traumatic symptoms [59]. A peculiarity of ICU-related PTSD is that patients are not affected by a single traumatic event but rather by continuous exposure to several traumatic episodes [60], altering the ability to process emotionally and integrate traumatic memory [61]. As a consequence, these fragmented memories of hearing conversations and sounds and feeling pain are often recalled and re-experienced as real [62]. Accordingly, early memories of the ICU stay are considered by different studies as predictors of PTSD symptoms onset [55,56]. The impact of these early memories on the onset of PTSD could explain why ICU diaries have proven of some benefit for ICU survivors, helping them to deal with their traumatic experiences [63].

### 3.4. Further Challenges for the ICU Survivors

As a consequence of the PICS-associated impairments, even patients functioning decently in their everyday lives may experience significant issues as having challenges in returning to their work, with the consequent financial burden for themselves, their families and society. Some authors have already associated the non-return to pre-existing employment with worsening cognition [64,65]. In a recent study, Mattioni et al. evaluated a Brazilian cohort of critically ill patients, reporting that over 50% were unable to return to their work within the first 3 months after ICU discharge. Some factors appear to increase the risk of being joblessness after ICU discharge, and they could be summarized as follows: pre-ICU factors (low educational level and being a regular employee), hospitalization-related factors (severity of the disease, need for MV), and post-ICU factors (physical limitations after discharge) [66]. Skei et al. are pioneers in this field being the first group to use nationwide registries to estimate the return to work in septic patients [67]. Their results show that 50% of ICU septic patients had resumed work by 2 years after their discharge. In terms of trajectory, a higher percentage of patients returned to work by one year as compared to those returning within 6 months or between the first and the second year after ICU discharge. Unsurprisingly, the authors reported better outcomes in younger patients and in those with fewer comorbidities and experiencing lower degrees of organ dysfunctions during the ICU stay. It is worth noting that the authors found no improvement in return to work between the first and the second year after ICU discharge, supporting the idea that there is a lack of rehabilitation and targeted interventions to improve long-term outcomes [67]. In a systematic review, Kamdar et al. highlighted that delayed return to work is common after critical illness, affecting around two-thirds, two-fifths, and one-third of previously employed survivors up to 3-, 12-, and 60 months following hospitalization, respectively [68]. It must be also considered that even for those able to return to work, a fair proportion of previously employed survivors require new disability benefits, in some instances associated with a substantial decrease in their earnings; further, they are vulnerable to occupation changes, job loss, and worsening of their employment status [68].

## 4. Post-Intensive Care Syndrome Family

The growing number of ICU survivors has resulted in an increased requirement for their care following the hospital discharge, which in most cases represents a burden for their families. In fact, more than half of survivors who received prolonged MV during their ICU stay will need assistance from a caregiver one year after ICU discharge [69]. Although caregiver assistance could be beneficial for the patient, such a burden may have negative consequences for the caregivers themselves, increasing their risk of mental health morbidity, with greater chances of developing anxiety, depression, and PTSD. This cluster of family complications in response to a recovery from critical illness of their loved one has been finally acknowledged with full dignity and termed as “post-intensive care syndrome—family” (PICS-F) [70,71].

Depression remains the most commonly investigated and reported psychological outcome across studies focusing on caregivers of ICU survivors. Current research suggests that risk factors for PICS-F include female gender and younger age. Other elements (i.e., social roles and cultural beliefs) may contribute to a predisposition for depression since it is frequent that females adopt caregiving roles within the family unit. The demand to cover these traditional roles may place females at greater risk for depression when there is further stress, such as the extra duty of caring for a recovering ICU survivor; such considerations have led to the hypothesis of “role strain” [72]. Interestingly, the institutionalization of ICU survivors in healthcare facilities after hospital discharge also seems associated with an increased risk of depressive symptoms in the caregivers. Such a finding suggests that the institutionalization of a loved ICU survivor could contribute to the onset of depressive symptoms, possibly due to the sense of guilt that the patient could not be cared for at home [73]. In general, caregivers may suffer not only from the burden of caring for the ICU survivor, but they usually experience interference with a significant reorganization of their lifestyle, adapting, for instance, their activities to the timing allowed for patient visitation and discussion with the medical team [74]. Since females are more likely to hold certain other responsibilities, such as dealing with children’s activities, it seems rather expected that the female gender represents a risk factor for depression in caregivers of ICU survivors [72].

A very important aspect of the PICS-F is the possibility that it develops also in those experiencing the loss of their loved one after ICU admission. Indeed, these individuals could be affected by prolonged grief due to a feeling that they should have done something differently before or during the hospital admission of their loved one. Such feeling could impact their mental health, hesitating into a dysfunctional syndrome characterized by the persistent focus on the loss of their loved one, the rumination about death, the inability to reorganize their life without their loved one, and the loss of any prospect of joy and happiness. Interestingly, it is estimated that such psychological disturbances have an overall prevalence of around 10% in families of bereaved people, and more than 50% among the relatives of patients dying in the ICU [75].

Looking at the management of PICS-F prevention, Kotfis et al. found a higher incidence of psychiatric symptoms in families of patients diagnosed with delirium during their ICU stay. This could be a further hint for medical staff on the importance of paying attention to delirium treatment/prevention, and assistance of caregivers of patients who suffered delirium [76]. Interestingly, a recent study by Dubont et al. tried to understand if a machine learning approach may help in predicting the odds of developing PTSD in patients’ family members [77]. Machine learning showed similar prediction power as compared to traditional linear statistical models, but it also took into consideration some hidden factors that influence the final results, opening a window to the opportunity to use the power of artificial intelligence. In future, healthcare providers could identify, prevent, and finally manage PTSD more easily, providing better support and improving the overall well-being of family members affected [77].

In order to reduce the burden of PICS-F, research has been developed around strategies to improve the overall ICU experience of the patients’ families. For example, the use of informational leaflets could be beneficial, facilitating the comprehension of the ICU environment and the consequences of the ICU stay [78]. The involvement of relatives in the patient’s therapeutic journey has been studied and it may also have positive effects. In a study on cardiac arrest patients, PTSD-related symptoms were less common in the family members who were given the opportunity to directly observe the cardiopulmonary resuscitation [79]. Moreover, a proactive communication strategy consisting of longer discussions with the family members seems to be associated with a decreased prevalence of symptoms of anxiety, depression, and PTSD [80]. A large trial demonstrated how an open ICU project allowing flexible visitation policies was associated with a decreased prevalence of anxiety and depression [81]. A recent study evaluated the use of facilitators to support communication between clinicians and families and showed that symptoms of depression were less common in family members when a facilitator was involved in the discussion [82]. Cox et al. compared the effects of two different interventions, a “coping skills training” and an education program on patient and family psychological distress. Despite the coping training not being superior in overall efficacy, it improved symptoms of distress at 6 months among patients with high baseline distress [83]. In a recent trial, Nielsen et al. studied the effect of a family-authored diary, showing its positive effects with a reduction in the risk of PTSD symptoms in the relatives, though without effects on depression, anxiety, or HRQOL [84].

## 5. Diagnostic Evaluation

There is no consensus on the best instruments for the evaluation and diagnosis of PICS. Spies et al. ideated a two-step assessment based on an initial screening that could be performed by any healthcare professional, which, in case of concern, is followed by a more comprehensive tool that should be performed by a PICS expert [85]. The initial screening consists of four tests for the screening of the following:

- Mental health: Patient Health Questionnaire-4;
- Cognition: MiniCog, Animal Naming;
- Physical function: Timed Up-and-Go, handgrip strength;
- HRQOL: EQ-5D-5L questionnaire.

The extended assessment is implemented in patients reporting new or worsening health problems after their ICU discharge or showing any abnormality in at least one of the mentioned screening tests, and consists of several tools:

- Mental health: Patient Health Questionnaire-8, Generalized Anxiety Disorder Scale-7, Impact of Event Scale—revised;
- Cognition: Repeatable Battery for the Assessment of Neuropsychological Status, Trail Making Test A and B;
- Physical function: 2-Minute Walk Test, handgrip strength, Short Physical Performance Battery;
- HRQOL: EQ-5D-5L, 12-Item WHO Disability Assessment Schedule [85].

Choosing the more suitable tool for a comprehensive PICS evaluation in all its domains represents a challenge. A scoping review analyzed 44 studies, and the authors found that only five tools accounted for all three domains of PICS [86]. In general, several problems persist in the diagnosis of PICS, which could be summarized as follows:

- A limited number of tools covering all the three domains of PICS;
- The unclear validity, and often limited feasibility, of these tools (i.e., translation);
- A low degree of evidence on the efficacy of these assessment tools on psychological health;
- Only two tools address the issue of PICS-F [86].

## 6. PICS Prevention and Treatment

**Figure 1:**
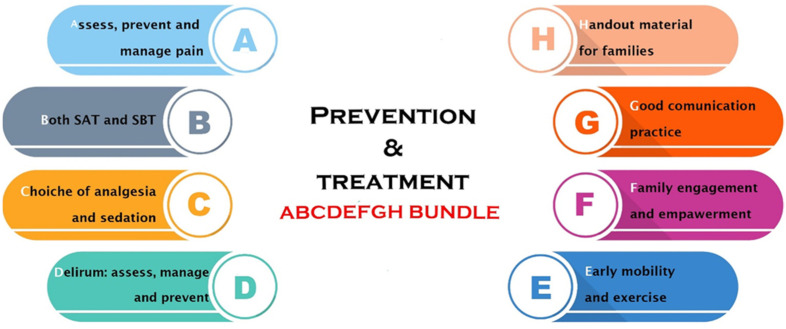
The “ABCDEFGH” bundle. The bundle is an evidence-based, multicomponent set of intensive care unit (ICU) interventions that could be applied to most patients and could have the value of reducing the burden of post-intensive care syndrome. SAT: spontaneous awakening trial; SBT: spontaneous breathing trial.

The growing interest in PICS has brought to the development of guidelines regarding the prevention and treatment of this condition. Such recommendations have been summarized in the “ABCDEFGH” bundle, which represents an evidence-based, multicomponent set of ICU interventions that may be potentially advantageous in reducing the burden of PICS. The bundle is schematically summarized in Figure 1 [87]. The implementation of this bundle can be useful for several reasons. First of all, it can be applied to every ICU patient every day during the ICU stay, regardless of the MV support or the admitting diagnosis. Second, this set of interventions is based on the assessment, prevention, and management of symptoms rather than on the disease processes itself. Third, the bundle is already relevant in the early period after ICU admission, and it does not interfere with ongoing life-sustaining therapies.

In general, the “ABCDEFGH” bundle has the ultimate goal of maximizing the number of arousable/awake patients, to make them cognitively engaged and physically active, as much as possible. In turn, this approach facilitates the capability of each patient to express unmet physical, emotional, and spiritual needs [87]. The single components of the bundle can be synthesized as follows:

A. Assessing and managing pain by using validated tools; pain management can be either pharmacological or non-pharmacological.
B. Breathing spontaneously should be encouraged with spontaneous breathing trials implemented unless contraindicated whilst correcting communication barriers.
C. Choice of the sedation, avoiding as much as possible benzodiazepines, using the allowed minimum dose of sedatives to avoid deep levels of sedation, and performing sedation holds as much as possible.
D. Delirium assessment on a daily basis in order to intervene as soon as possible both pharmacologically and non-pharmacologically.
E. Early mobilization with a physiotherapy program to reduce the physical decline and the loss of muscular mass; the use of electrical muscle stimulation seems quite promising [88,89].
F. Family involvement by the healthcare providers, enhancing communication strategies.
G. Good communication practices with the family members from the medical team to prevent the PICS-F.
H. Hand out material provided to families to allow a better understanding of the ICU environment.

Other interventions that could contribute to reducing the burden of PICS have been proposed, and include avoiding hypoglycemia and hypoxemia to reduce the incidence of delirium and encephalopathy; introducing ICU diaries that may decrease the PTSD symptoms and could have beneficial effects for the family members; creating post-ICU clinics to guarantee a proper patient’s follow-up and counseling for the survivors and their families [90,91].

Apart from preventive measures, treatment options should be considered. Patients with psychiatric symptoms may benefit from the treatment with a combination of pharmacotherapy and also non-pharmacological, psychological, and behavioral therapies. A recent study in a murine model suggested that the GABAergic system may play a significant role in the pathophysiology of PICS and that early treatment with fluoxetine may alleviate symptoms [92]. However, the first-line non-pharmacological treatment remains cognitive-behavioral therapy, with the great advantage that nowadays this intervention is deliverable via smartphones and computer applications [93]. Another issue in patients developing PICS is their need for polytherapy, with several medications started during the ICU stay and continued after discharge. Apart from the psychological impact of such a polytherapy, these drugs may produce side effects, from minor to clinically major ones, in turn worsening the patient’s QOL. In order to avoid these issues, integrating pharmacists into a multidisciplinary follow-up team has been proposed [94]. This approach may decrease medication-related issues [95]. In order to guarantee a continuum of care from the inpatient to the outpatient setting, a valuable option could be to involve the same pharmacist dealing with the patient during the ICU stay [96]. 

The rehabilitation process after ICU and hospital discharge may certainly benefit from multidisciplinary team support, including not only a physician from the ICU team, but also a rehabilitation team (including physicians, nurses, psychologists, physical therapists, and occupational therapists) to cover all the aspects of recovery. Indeed, the complexity of PICS certainly suggests a need for individual multidisciplinary, and multi-professional support to enhance recovery and rehabilitation from motor, cognitive, and psychological health perspectives [97]. Indeed, to regain all physical and cognitive functions as soon as possible, patients would benefit from physical therapy, respiratory muscle training, swallowing exercises, psychological support, occupational therapy, and cognitive care. Interestingly, in an observational study, Peris et al. showed the importance of implementing early intra-ICU support by clinical psychologists. In a population of critically ill trauma patients, the authors showed that patients exposed to clinical psychologists halved the risk of anxiety and depression (though the result was statistically non-significant), significantly reduced the risk for PTSD, and showed a lower prevalence of using psychiatric medications at 12 months; these results suggest that it could be valuable to start psychologist support as soon as possible after ICU admission [98]. Yasaka et al. evaluated the effect of early rehabilitation programs promoted by the Japanese government with financial incentives to hospitals, showing a positive outcome for patients and highlighting again the importance of early intervention [99]. Moreover, the early introduction of mobilization has been studied. Schweickert et al. showed that the patients randomized to receive physical and occupational therapy starting within 72 h of ICU admission regained their independence at ICU discharge sooner than the control group; indeed, the intervention group had improved functional status, lower incidence of ICU and hospital delirium, and shorter duration of MV [100]. In order to promote the patient’s recovery, physical rehabilitation can be also implemented with innovative techniques such as neuromuscular electrical stimulation (NMES) and inspiratory muscle training (IMT). The NMES can be applied to prevent ICUAW, and a meta-analysis suggested that it could be effective in increasing muscle strength and reducing the duration of MV and the ICU stay [101]. Moreover, when associated with physical therapy, NMES significantly increases the success of extubation [102]. The IMT is another emerging supportive approach aiming at reducing diaphragmatic dysfunction in ICU patients. It can be achieved with a wide range of techniques such as portable devices or through the ventilator’s triggering settings. A meta-analysis suggests that IMT applied to ICU patients undergoing MV could be effective in improving the weaning process from MV [103].

Another fundamental point regarding the patient’s rehabilitation is the nutrition after ICU discharge. Notably, pre-admission nutritional status seems a key determinant for the outcome of ICU patients, and this negative factor does not seem to improve when a high protein intake during ICU stay is provided [104]. Moreover, another randomized evidence has suggested that, when compared to standard enteral protein provision (1.3 g/kg/day), a high enteral protein regimen (2.0 g/kg/day) resulted in worse HRQOL in ICU patients, without improving functional outcomes 6 months after admission [105]. However, some authors suggest that the ideal caloric intake could be higher during the recovery from critical illness and after ICU discharge (35 kcal/kg/day with 2.0–2.5 g/kg/day of protein load). High-dose protein and calorie feeding for a prolonged duration after ICU discharge might be necessary to optimize the patient’s outcome, and pharmacological options and oral supplements could help in achieving these goals. Nevertheless, the authors acknowledge that achieving such protein targets could be a real challenge. [106]. However, nutritional intake after ICU is often decreased for several reasons, including psychological aspects. Dysphagia may affect up to 80% of ICU patients, causing a permanent disability in almost 60% of them [30,107]. Notably, there is a lack of evidence about both the real energy expenditure after critical illness and the effectiveness of nutritional interventions after hospital discharge. In a small study, Salisbury et al. found no difference between patients with or without nutrition rehabilitation [108]. Meanwhile, it seems reasonable to involve dieticians in the rehabilitation process after ICU discharge in order to achieve optimal nutritional requirements [109].

## 7. Conclusions

In this narrative review, we attempted to comprehensively summarize the knowns and the unknowns of PICS while also digging into PICS-F. Both conditions are more complex syndromes than was previously thought, with a significant concurrence of physical, psychological, cognitive, and social impairments that may last long after the critical illness. 

Despite the social relevance of PICS and PICS-F and their consequences, there is a huge knowledge gap in the understanding of its pathophysiology and, consequently, in the possible strategies for the prevention and treatment of these syndromes. Moreover, the challenges in producing a precise quantification of the financial burden arising from these conditions make it even more complex to convince the stakeholders of the importance of investing in the prevention of PICS and PICS-F, and/or in investing in ICU follow-up clinics. 

## Figures and Tables

**Table 1:** Risk factors potentially associated with the development of post-intensive care syndrome. BMI: body mass index; ICU: intensive care unit; LOS: length of stay.

ICU related	Admission	Emergency admission, ICU type, hospital type, ICU LOS, and Hospital LOS
	Experience	ICU mentality, delirium, restraint, bed rest, device self-removal, pain
	Treatment and therapies	Diagnosis, comorbidity, surgery, complications, disease severity, type of support (cardiovascular, respiratory, renal), no. of organs supported, analgesics, drugs administered (muscle relaxants, sedatives, steroids, inotropic drugs), no. of drug groups, laboratory data, vital signs
Patient’s related	Personality traits	Illness awareness, mindfulness, optimism, coping skill, self-efficacy, and trait anxiety
	Previous health conditions	BMI, hearing or visual impairment, previous ICU admission, pre-ICU sleep quality, frailty, trauma event, mental health problem, cognitive function, physical status
	Social and demographics	Age, sex, ethnicity, living situation, marital status, younger children, education, employment, socioeconomic status, caregiver, social support, social issue, alcohol, smoking, illicit drug, and physical activity

## Data Availability

Not applicable, but corresponding author happy to be reached for further information.
